# Immunoglobulin characteristics and RNAseq data of FcRL4+ B cells sorted from synovial fluid and tissue of patients with rheumatoid arthritis

**DOI:** 10.1016/j.dib.2017.06.009

**Published:** 2017-06-07

**Authors:** Khaled Amara, Elizabeth Clay, Lorraine Yeo, Daniel Ramsköld, Julia Spengler, Natalie Sippl, James Cameron, Lena Israelsson, Philip J. Titcombe, Caroline Grönwall, Ilfita Sahbudin, Andrew Filer, Karim Raza, Vivianne Malmström, Dagmar Scheel-Toellner

**Affiliations:** a Rheumatology Unit, Department of Medicine, Karolinska University Hospital, Karolinska Institutet, SE-17176 Solna, Stockholm, Sweden; b Rheumatology Research Group, RACE AR UK Centre of Excellence in RA Pathogenesis, Institute of Inflammation and Ageing, College of Medical and Dental Sciences, University of Birmingham, Birmingham B15 2TT, UK; c Department of Rheumatology, Sandwell and West Birmingham Hospitals NHS Trust, Birmingham, UK

**Keywords:** B cells, FcRL4, Rheumatoid arthritis, IRTA1

## Abstract

This manuscript is a companion paper to Amara et al. [1]. Data shown here include detailed clinical characteristics from anonymized patients, the Ig subclass data generated from B cells sorted from four individual patients, tables detailing variable gene region sequences from sorted cells linked to the patient information and the sequence yields from individual patients. Furthermore a URL link to the RNAseq datasets submitted to GEO is included.

**Specifications Table**

**Table:** 

Subject area	Immunology
More specific subject area	B cells in Rheumatoid Arthritis
Type of data	1 figure, 3 tables, url to data
How data was acquired	Sequencing, patient clinical characteristics linked to experimental data
Data format	Analyzed, raw
Experimental factors	FcRL4+ and FcRL4- B cells were sorted from synovial fluid and tissue from RA patients. Synovial fluid derived B cells were analysed for their gene expression profile by RNAseq. Immunoglobulin variable region genes from single sorted B cells were sequenced and expressed as components of recombinant monoclonal antibodies. These were investigated for their reactivity with autoantigens.
Experimental features	Data shown here include detailed clinical characteristics from anonymized patients, the Ig isotype data generated from B cells sorted from four individual patients, tables detailing variable gene region sequences from sorted cells linked to the patient clinical characteristics and the sequence yields from individual patients. We also supply a URL link to the RNAseq datasets submitted to GEO.
Data source location	Birmingham UK
Data accessibility	https://www.ncbi.nlm.nih.gov/geo/query/acc.cgi?acc=GSE94897
Related research article	1) Amara K, Clay E, Yeo L, Ramsköld D, Spengler J, Sippl N, Cameron JA, Israelsson L,Titcombe PJ, Grönwall C, Sahbudin I, Filer A,Raza K, Malmström V, Scheel-Toellner D.
	B cells expressing the IgA receptor FcRL4 participate in the autoimmune response in patients with rheumatoid arthritis. J Autoimmun. 2017. pii: S0896-8411(16)30396-1. doi: 10.1016/j.jaut.2017.03.004 [Epub ahead of print]

**Value of the data**

• First RNAseq dataset from FcRL4+ and FcRL4- B cells sorted from the synovial fluid of patients with rheumatoid arthritis. This will be valuable to researchers interested in the regulation of B cell subpopulations and their functional role in RA.
• Ig subclass distribution in FcRL4+ and FcRL4- B cells infiltrating the rheumatoid joint. This gives important information of the potential origins of these cells and their potential function in the joint.
• The tables linking variable region sequences, gene usage, Ig isotypes and reactivity with citrullinated autoantigens give insight into the immune response to citrullinated proteins on a single cell basis.

## 1 Data

**Fig. 1:**
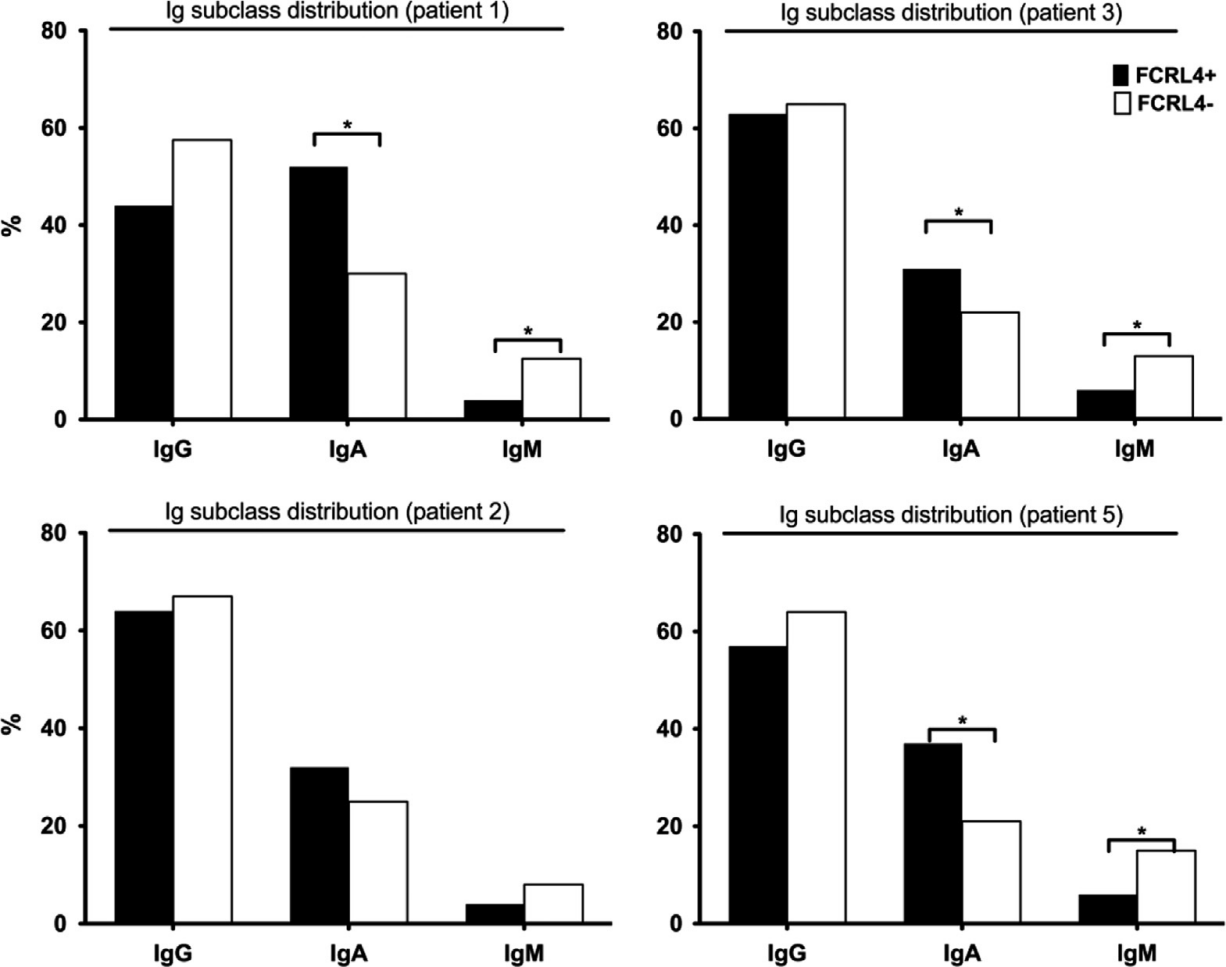
Ig subclass distribution determined in single sorted FcRL4+ and FcRL4- B cells in four individual patients.

The data shown in this manuscript have been generated in a study of FCRL4+ and FcRL4- B cells infiltrating the synovial fluid and synovial tissue of RA patients. They include a link to the GEO dataset of RNAseq gene expression profiles of these cells. Furthermore, the Ig isotype distribution of the B cells for these populations is shown for four individual patients in Fig. 1. Table 1 gives detailed clinical characteristics from the anonymized patients. These are linked to the data shown in Table 2, detailing variable gene region sequences from sorted cells, the isotype usage and reactivity with citrullinated proteins of these individual cells. Table 3 displays the number of sequences and recombinant monoclonal antibodies generated from FcRL4+ and FcRL4- B cells from individual patients.

**Table 1:** Clinical characteristics of RA patients who provided synovial fluid or synovial tissue. RF, rheumatoid factor; CCP, cyclic citrullinated peptide; CRP, C reactive protein; ESR, erythrocyte sedimentation rate, DAS28, disease activity score 28, TJC, tender joint count, SJC swollen joint count, VAS visual analog score. Hydroxychlor., Hydroxychloroquine. NA not available.

**Patient identifier**	**Sample**	**Diagnosis**	**Gender**	**Age (yrs)**	**Age at onset of RA**	**Dis. Dur. (yrs)**	**RF**	**CCP**	**CRP**	**ESR**	**DAS 28 ESR**	**TJC (28)**	**SJC (28)**	**VAS gen. health**	**Figures**	**Current disease modifying therapy**
1	ST	RA	F	69	27	42	NA	pos	0	18	5.6	12	3	80	1A, 1B, 2A, 2B, 3A, 3B, 3C, 4A, 4B	Adalimumab
2	ST	RA	M	70	55	15	pos	pos	11	24	3.86	2	1	40	1A, 1B, 2A, 2B, 3A, 3B, 3C	Etanercept, Methotrexate, Hydroxychlor.
3	SF	RA	M	72	72	0.23	neg	neg	23	30	6.0	11	14	49	1A, 1B, 2A, 2B, 3A, 3B, 3C	Nil
4	SF	RA	F	52	41	12	neg	neg	0	38	5.4	5	2	89	1A, 1B, 2A, 2B, 3A, 3B, 3C	Methotrexate Tocilizumab
5	SF	RA	F	40	40	0.23	pos	pos	8	9	5.9	19	8	84	1A, 1B, 2A, 2B, 3A, 3B, 3C, 4A, 4B	Hydroxychlor.
6	SF	RA	F	49	38	11	pos	na	3	15	3.1	2	2	0	1A, 1B, 2A, 2B, 3A, 3B, 3C	Etanercept, Methotrexate
7	SF	RA	M	68	56	12	NA	NA	19	15	NA	NA	NA	NA	1C	Methotrexate
8	SF	RA	F	60	51	9	NA	pos	45	13	NA	6	NA	NA	1C	Methotrexate
9	SF	RA	F	44	41	3	neg	neg	13	29	4.95	7	3	45	1C	Methotrexate
10	SF	RA	F	60	57	3	pos	pos	20	55	7.05	15	10	85	1C	Nil
11	SF	RA	M	39	34	5	NA	NA	NA	NA	NA	NA	NA	NA	1C	Etanercept
12	SF	RA	F	70	68	2	pos	neg	142	21	4.8	5	2	73	1C	Prednisolone
13	SF	RA	F	60	57	3	pos	pos	122	104	5.88	2	4	91	1C	Sulfasalazine
14	SF	RA	F	60	29	31	neg	pos	57	28	6.75	16	11	89	1C	Prednisolone
15	SF	RA	F	63	62	1	pos	pos	30	60	4.13	1	2	22	1C	Methotrexate, prednisolone
16	SF	RA	M	54	54	0.23	neg	neg	88	58	7.39	23	12	93	1C	Nil
17	SF	RA	F	77	68	9	neg	neg	71	62	NA	NA	NA	NA	1C	Methotrexate, Prednisolone
18	SF	RA	M	72	56	16	pos	pos	5	15	13	NA	NA	NA	5A, 5B	Etanercept, Methotrexate, Hydroxychlor.
19	SF	RA	F	32	31.2	0.8	pos	pos	15	48	5.9	8	6	93	5A, 5B	Nil
20	SF	RA	F	52	49	3	pos	pos	13	34	6.54	14	10	78	5A, 5B	Nil
21	SF	RA	F	46	35	9	pos	pos	NA	NA	NA	NA	NA	NA	5A, 5B	Methotrexate, Hydroxychlor.

**Table 2:** Sequence data and reactivity of monoclonal antibodies from FcRL4+ and FcRL4- B cells from RA patients.

**Patient id**	**Clone**	**cell origin**	**VH**	**DH**	**JH**	**Ig SC**	**VH-mut.**	**VH CDR3 (aa)**	**(+)**	**Length**	**CCP2**	**CEP-1**	**cit-vim 60-75**	**cit-fib 36-52**
1	146+.A06	ST (CD19+FcRL4+)	3-33	2-2	6	IgG1	6	SRVGRVPDAVRYYFYGDV	3	18				
	146+.A07	ST (CD19+FcRL4+)	3-15	2-2	6	IgG1	29	ATDVFRTVVPVAIYSFYGLAV	1	21	**pos**	**pos**	**pos**	neg
	146+.A08	ST (CD19+ FcRL4+)	3-11	4-17	4	IgA1	10	ARGRWGLYGDYIFDS	2	15				
	146+.A09	ST (CD19+ FcRL4+)	5-51	3-22	3	IgA1	1	ARPHYYDSLDAFDI	2	14				
	146+.A10	ST (CD19+ FcRL4+)	3-33	6-13	4	IgA1	10	AREEGTGIVATDTESDFFDS	1	20	neg	neg	neg	neg
	146+.A11	ST (CD19+ FcRL4+)	1-18	3-10	5	IgA1	15	ARRPDSSQYSNWIDP	2	15	neg	neg	neg	neg
	146+.A12	ST (CD19+ FcRL4+)	3-23	6-13	5	IgA1	23	AKDDSPIATHSSWDS	2	15	neg	neg	neg	neg
	146+.B01	ST (CD19+ FcRL4+)	3-30-3	3-22	4	IgG1	35	VRGYCSSLSCSSFDS	1	15	neg	neg	neg	neg
	146+.B02	ST (CD19+ FcRL4+)	1-46	3-3	3	IgA1	20	ARAEGAVTIDDAFDI	1	15	neg	neg	neg	neg
	146+.B03	ST (CD19+ FcRL4+)	1-69	2-8	4	IgG1	17	ARHCNNGLCFYYFDY	2	15				
	146+.B04	ST (CD19+ FcRL4+)	3-30	4-11	4	IgG1	18	VKDAYRTQSPHFNNR	4	15				
	146+.B05	ST (CD19+ FcRL4+)	4-59	6-13	6	IgA1	21	ARAGSWFLYGMDV	1	13				
	146+.B06	ST (CD19+ FcRL4+)	4-4	3-10	3	IgG1	8	TSPQGGPGSYPHDAFDV	1	17	neg	neg	neg	neg
	146+.B08	ST (CD19+ FcRL4+)	1-2	3-9	4	IgG1	8	CARLRQDFDLLTGYQLGSYYFDY	2	23	neg	neg	neg	neg
	146+.B12	ST (CD19+ FcRL4+)	1-18	1-26	6	IgG3	26	AKDQWEAYYGLDV	1	13				
	146+.C01	ST (CD19+ FcRL4+)	3-23	2-2	6	IgA1	12	ARGKARYQLPTYFYYGMDV	3	19	neg	neg	neg	neg
	146+.C04	ST (CD19+ FcRL4+)	3-53	3-3	4	IgG1	14	ARAAVDFWSGYHLEY	2	15				
	146+.C05	ST (CD19+ FcRL4+)	5-51	6-13	6	IgA2	22	TRLGSWYLHYYGVDV	2	15				
	146+.C06	ST (CD19+ FcRL4+)	3-23	2-15	6	IgA1	15	AKRVVVASNHGYYSMDV	3	17				
	146+.C08	ST (CD19+ FcRL4+)	3-21	3-9	4	IgA1	20	ARDYDVFTGYPSHFFDH	3	17				
	146+.C09	ST (CD19+ FcRL4+)	3-21	2-15	6	IgG2	25	ARDRVDIVVKEPNFYYGVDV	3	20				
	146+.C10	ST (CD19+ FcRL4+)	4-34	1-26	4	IgA1	29	ANRRRYTTRFYFDF	4	14				
	146+.D07	ST (CD19+ FcRL4+)	3-23	3-10	1	IgA1	1	AKDRGVLRYFDWLH	4	14	neg	**pos**	**pos**	neg
	146+.D08	ST (CD19+ FcRL4+)	4-30-4	3-3	5	IgG2	36	ASSGFFGQPYNWFDR	1	15				
	146+.D09	ST (CD19+ FcRL4+)	3-48	3-3	4	IgG1	31	ARDTRDFWSGYYTYYFDY	2	18	pos	neg	neg	neg
	146+.D10	ST (CD19+ FcRL4+)	5-51	4-23	6	IgA1	19	ARLGKTTTVTSPYYYYYGMDV	2	21	neg	neg	neg	neg
	146+.E01	ST (CD19+ FcRL4+)	1-69	5-12	4	IgG1	12	ARDSGYDEGYYFDY	1	14	neg	neg	neg	neg
	146+.E02	ST (CD19+ FcRL4+)	3-9	1-26	4	IgA1	24	AKASGLTGSFYPLDH	2	15				
	146+.E04	ST (CD19+ FcRL4+)	4-34	2-15	4	IgG1	6	ARGGHRKYCSGGSCIYYFDY	4	20				
	146+.E05	ST (CD19+ FcRL4+)	3-11	5-12	3	IgA1	19	ARDDTVAFKDALDI	2	14	neg	neg	neg	neg
	146+.E06	ST (CD19+ FcRL4+)	1-69	1-1	4	IgA1	34	ARERALCAEGCPPGDD	2	16	neg	**pos**	neg	neg
	146+.E07	ST (CD19+ FcRL4+)	5-10-1	3-10	4	IgA2	16	ARVRTYYSAGTYPFDS	2	16				
	146+.E08	ST (CD19+ FcRL4+)	3-30	2-8	6	IgA1	21	AKAWGQLAGFALYFYGLDV	1	19				
	146+.E12	ST (CD19+ FcRL4+)	3-48	3-3	4	IgA1	26	ARINYDYWSDYARFLDS	2	17				
	146+.F01	ST (CD19+ FcRL4+)	4-30-4	3-10	4	IgG1	0	AFHLGEYGSGSYYDLDY	1	17	neg	neg	neg	neg
	146+.F02	ST (CD19+ FcRL4+)	4-38-2	6-13	3	IgA1	31	ARDRGYSTNWFLGFDV	2	16	neg	neg	neg	neg
	146+.F04	ST (CD19+ FcRL4+)	3-21	2-8	4	IgG1	19	AKPTVVYGPIDY	1	12				
	146+.F08	ST (CD19+ FcRL4+)	4-38-2	3-3	4	IgA1	22	AREFEHFGSGYFPVDY	2	16				
	146+.F09	ST (CD19+ FcRL4+)	3-49	3-3	4	IgA1	26	NSRSGFGVVAPEIDH	2	15				
	146+.F10	ST (CD19+ FcRL4+)	3-23	1-26	4	IgA1	20	ATDGEGVLFDE	0	11	neg	neg	neg	neg
	146+.F11	ST (CD19+ FcRL4+)	4-59	3-3	3	IgA1	22	ARVMTVFGVVPDAFDI	1	16				
	146+.F12	ST (CD19+ FcRL4+)	3-43	6-19	4	IgM	3	AKDISSTGWEYCFEN	1	15				
	146+.G01	ST (CD19+ FcRL4+)	3-20	1-26	4	IgA1	15	AKPSRVGAAADADY	2	14				
	146+.G02	ST (CD19+ FcRL4+)	4-31	3-3	5	IgG1	10	AREGVHATTFGMIDDQGWFDP	2	21	neg	neg	neg	neg
	146+.G03	ST (CD19+ FcRL4+)	1-46	1-14	6	IgG1	18	ARVSPGIRDDMDV	2	13	neg	neg	neg	neg
	146+.G04	ST (CD19+ FcRL4+)	3-30	7-27	4	IgG1	4	ARESGARWDVYFDY	2	14	neg	neg	neg	neg
	146+.G05	ST (CD19+ FcRL4+)	4-30-4	3-16	5	IgG1	36	ARAPPETLRGIVGNWFDP	2	18	neg	**pos**	**pos**	neg
	146+.G06	ST (CD19+ FcRL4+)	1-3	2-15	4	IgG1	14	VKDGGAGGANTFDH	2	14				
	146+.G07	ST (CD19+ FcRL4+)	3-33	5-24	4	IgA1	31	ARARRGDGYNQARYYYFDY	4	19	neg	**pos**	neg	neg
	146+.G09	ST (CD19+ FcRL4+)	1-18	6-19	3	IgA1	4	ARGWYSRGGGMDV	2	13	neg	neg	neg	neg
	146+.G10	ST (CD19+ FcRL4+)	1-18	3-16	4	IgG1	10	ARGWDPIVLPDYW	1	13				
	146+.G11	ST (CD19+ FcRL4+)	3-23	2-2	4	IgA1	27	AKSHLAHYVPVPAPFDF	3	17				
	146+.G12	ST (CD19+ FcRL4+)	1-3	1-26	6	IgM	1	TRDLLDRGKYYRVAGHFYGMDV	5	22				
	146+.H02	ST (CD19+ FcRL4+)	3-30	3-9	6	IgG3	33	ARDGGENEIVTGYFGWSNKPHSVKYYHGMDV	5	31				
	146+.H05	ST (CD19+ FcRL4+)	4-61	3-22	2	IgG2	3	ARHVGRLRRDSFTTRRTTDAADDWHIDL	8	28				
	146+.H07	ST (CD19+ FcRL4+)	3-30	1-26	5	IgG1	26	AKQSATMGPNRQPR	3	14	neg	neg	neg	neg
	146+.H08	ST (CD19+ FcRL4+)	4-34	6-13	5	IgA1	9	ARGFWDSGSWFDY	1	13				
	146+.H09	ST (CD19+ FcRL4+)	4-34	2-21	6	IgG1	8	ASKGGDSVGYHYYMDV	2	16	neg	neg	neg	neg
	146+.H10	ST (CD19+ FcRL4+)	1-18	3-3	5	IgA1	14	ARGRPSTFGVVRGFDP	3	16				
	146-.A03	ST (CD19+ FcRL4-)	3-30	4-17	4	IgG1	13	TRATRVNGNLNTFDY	2	15	neg	neg	neg	neg
	146-.A06	ST (CD19+ FcRL4-)	3-9	3-3	5	IgG1	14	AKDRFGELTDLTYVGWFDP	2	19	neg	neg	neg	neg
	146-.A07	ST (CD19+ FcRL4-)	5-10-1	3-10	5	IgA1	31	ARLDTSVIRGYNWFDP	2	16	neg	neg	neg	neg
	146-.B05	ST (CD19+ FcRL4-)	4-59	2-15	3	IgA1	7	ARHRGGSPTAFDI	3	13	neg	neg	neg	neg
	146-.B07	ST (CD19+ FcRL4-)	3-15	4-17	4	IgG1	17	TTVDDYECHDY	1	11				
	146-.B11	ST (CD19+ FcRL4-)	4-4	2-15	4	IgG1	17	ARVSEAYFDPFYYDNN	1	16				
	146-.B12	ST (CD19+ FcRL4-)	1-18	3-9	4	IgG1	30	ARAPGSLRYYDWVSLYEEGDH	3	21	neg	neg	neg	neg
	146-.C01	ST (CD19+ FcRL4-)	1-46	3-10	3	IgA1	20	TSPQGGPGSYPHDAFDV	1	17	neg	neg	neg	neg
	146-.C07	ST (CD19+ FcRL4-)	1-46	3-3	6	IgG1	29	ARVTTFESGPNDFGVPDHFYYVLDV	2	25	neg	neg	neg	neg
	146-.C10	ST (CD19+ FcRL4-)	4-34	1-26	4	IgA1	32	ANRRRCTTRFYFDF	4	14	neg	neg	neg	neg
	146-.D03	ST (CD19+ FcRL4-)	3-23	2-8	4	IgG1	22	AKPLVYARLYFYYDLDY	2	17	neg	neg	neg	neg
	146-.D05	ST (CD19+ FcRL4-)	3-48	3-22	4	IgG1	0	VRDSPGWGFRYYDY	2	14	neg	neg	neg	neg
	146-.D06	ST (CD19+ FcRL4-)	4-30-4	3-22	4	IgM	3	AAYPGDNSGRHLISPPFDN	2	19				
	146-.D09	ST (CD19+ FcRL4-)	4-30-4	3-22	4	IgG1	26	AAYGSYDRHLISPNPFYYDN	2	20	neg	neg	neg	neg
	146-.E04	ST (CD19+ FcRL4-)	3-7	6-13	6	IgG2	25	VSQQVVPC	0	8				
	146-.E05	ST (CD19+ FcRL4-)	3-7	6-13	6	IgA2	31	VSGGLQQYDVVPC	0	13				
	146-.E06	ST (CD19+ FcRL4-)	4-34	2-15	4	IgA1	8	ARGGHRKYCSGGSCIYYFDY	4	20	neg	neg	neg	neg
	146-.E07	ST (CD19+ FcRL4-)	3-74	2-2	6	IgG3	11	ARVQPQRVLVFYGMDV	2	16				
	146-.E08	ST (CD19+ FcRL4-)	3-11	4-17	4	IgG1	16	ARGAVTTPEYYFDY	1	14	neg	neg	neg	neg
	146-.E10	ST (CD19+ FcRL4-)	4-59	4-11	4	IgA1	25	ARLDYSPAFIFDS	1	13	neg	neg	neg	neg
	146-.E11	ST (CD19+ FcRL4-)	3-30-3	3-9	4	IgG1	13	ARGWERYYDWVAPGH	3	15				
	146-.F01	ST (CD19+ FcRL4-)	3-11	3-10	6	IgG4	26	ARGPSGMFGDLSPYFHYGVDV	2	21				
	146-.F04	ST (CD19+ FcRL4-)	5-10-1	2-15	5	IgG1	19	ARHGRGPSSWYDF	3	13				
	146-.F08	ST (CD19+ FcRL4-)	1-3	2-8	4	IgA1	25	ARSHQPYILLAGTPGD	2	16	neg	neg	neg	neg
	146-.F10	ST (CD19+ FcRL4-)	5-10-1	2-15	3	IgA1	19	AKAASRFDTFDI	2	12	neg	neg	neg	neg
	146-.F11	ST (CD19+ FcRL4-)	4-31	4-23	4	IgG2	22	TRGVIGLRGVPYYFDS	2	16				
	146-.G03	ST (CD19+ FcRL4-)	3-66		4	IgG1	17	RVDDTAVYYCARSPTGYDILTGPFDY	2	26				
	146-.G04	ST (CD19+ FcRL4-)	1-18	6-19	2	IgM	5	ARAVAVNWYFDL	1	12				
	146-.G05	ST (CD19+ FcRL4-)	3-48	5-18	3	IgM	0	ARGRKGYSYDAFDI	3	14				
	146-.G07	ST (CD19+ FcRL4-)	3-20	3-22	4	IgG1	12	ARGPPYYISSGYYFSFDS	1	18				
	146-.H01	ST (CD19+ FcRL4-)	4-59	2-21	4	IgA1	22	ARDDSLGGFDY	1	11	neg	neg	neg	neg
	146-.H02	ST (CD19+ FcRL4-)	3-23	3-22	5	IgM	2	AKYYDTSGSYKACDI	2	15				
	146-.H04	ST (CD19+ FcRL4-)	3-11	3-22	4	IgG1	25	ARGFYHDGTAYYHRNQSPFDH	5	21				
**Patient id**	**Clone**	**cell origin**	**VH**	**DH**	**JH**	**Ig SC**	**VH-mut.**	**VH CDR3 (aa)**	**(+)**	**Length**	**CCP2**	**CEP-1**	**cit-vim 60-75**	**cit-fib 36-52**
5	153+.A04	SF (CD19+ FcRL4+)	4-39	3-10	5	IgG1	6	ARLGGGYYYGSGYTRFDP	2	18	neg	**pos**	neg	neg
	153+.A08	SF (CD19+ FcRL4+)	3-13	3-9	1	IgA1	16	ATKPSHIYLRYFDWLLQGVRPLL	4	23				
	153+.A09	SF (CD19+ FcRL4+)	4-39	3-22	4	IgG1	2	ARYLREDYDISGLDY	2	15	neg	neg	neg	neg
	153+.A10	SF (CD19+ FcRL4+)	1-69	3-10	3	IgG1	12	ARRGYYYDYVWGDFRLTGPIEGAFDI	3	26	neg	neg	neg	nrg
	153+.B05	SF (CD19+ FcRL4+)	4-59	3-22	4	IgG3	13	AADNYYDSSEYSPYSFDS	0	18				
	153+.B08	SF (CD19+ FcRL4+)	3-48	3-3	6	IgG4	4	ASDKYDSWSRYVPYYGLDV	2	19				
	153+.C03	SF (CD19+ FcRL4+)	3-33	3-9	4	IgG2	8	ARGPDILTGGFYFDY	1	15				
	153+.C04	SF (CD19+ FcRL4+)	3-74	3-10	4	IgG1	6	VRGDLWFVELLYG	1	13	neg	neg	neg	neg
	153+.C06	SF (CD19+ FcRL4+)	1-18	3-22	4	IgA1	2	ARGSPYYYDSSGYYHYFDS	2	19	neg	neg	neg	neg
	153+.C09	SF (CD19+ FcRL4+)	4-59	3-3	6	IgA1	3	ARDKSADTLEWYYYYYGMDV	2	20				
	153+.C10	SF (CD19+ FcRL4+)	5-51	6-19	4	IgA1	4	APQSGSGWPYFDY	0	13				
	153+.C12	SF (CD19+ FcRL4+)	3-33	7-27	4	IgA1	16	ARHRGVTGLLNEPGDY	3	16	neg	neg	neg	neg
	153+.D01	SF (CD19+ FcRL4+)	3-33	2-21	4	IgA1	1	ARLLKTYCGGDCSLGY	2	16				
	153+.D06	SF (CD19+ FcRL4+)	5-51	3-3	6	IgA1	0	ARQYYDFWSDYYNSDYYYGMDV	1	22				
	153+.D08	SF (CD19+ FcRL4+)	4-34	3-10	6	IgA1	7	ARESHDHAELGYYYGMDV	3	18				
	153+.E01	SF (CD19+ FcRL4+)	4-59	6-13	4	IgA1	7	ASLPGSSTWFPFDY	0	14				
	153+.E03	SF (CD19+ FcRL4+)	3-21	3-10	4	IgA1	10	ARIRTKWFRRSSTMSSSFDY	5	20				
	153+.E04	SF (CD19+ FcRL4+)	1-69	2-15	6	IgG2	6	ARGRVPRIYYYYGMDV	3	16				
	153+.E05	SF (CD19+ FcRL4+)	1-2	3-22	3	IgG1	7	ARCDWGIYYYDSRAHGAFDF	3	20	neg	neg	neg	neg
	153+.E07	SF (CD19+ FcRL4+)	4-30-2	3-9	4	IgM	1	ARDQFFLAALDY	1	12				
	153+.E11	SF (CD19+ FcRL4+)	1-69	4-17	6	IgG2	4	AREDYGDDYYYYGMDV	1	16				
	153+.F02	SF (CD19+ FcRL4+)	3-66	3-22	3	IgG3	4	AREYNYDSSDAFDI	1	14				
	153+.F04	SF (CD19+ FcRL4+)	1-69	6-6	4	IgG2	5	ARSVQNLRYLGYYFDY	2	16				
	153+.F07	SF (CD19+ FcRL4+)	3-15	3-9	4	IgG1	5	TSSLVLRYFDWSTHSSDY	2	18	neg	neg	neg	neg
	153+.G01	SF (CD19+ FcRL4+)	3-15	1-1	4	IgG1	4	TTALNWNWDYYDY	0	13	neg	neg	neg	neg
	153+.G07	SF (CD19+ FcRL4+)	3-9	2-15	4	IgG2	9	VASYWRGYYFDY	1	12				
	153+.H01	SF (CD19+ FcRL4+)	3-33	3-3	6	IgG1	8	AKVAGYDFWSGPGGYYYSMDV	1	21				
	153+.H02	SF (CD19+ FcRL4+)	1-18	2-15	5	IgM	4	ARDSGGSWLDP	1	11				
	153+.H06	SF (CD19+ FcRL4+)	3-74	1-7	4	IgG1	7	ARGGWGPRYNWNQGAVDY	2	18	**pos**	**pos**	**pos**	neg
	153+.H11	SF (CD19+ FcRL4+)	3-11	4-11	6	IgA1	18	ARQSAYANYYYKGMDV	2	16				
	153-.A03	SF (CD19+ FcRL4-)	4-34	3-22	2	IgA1	14	ARGLTFSYYDSSGFGYYYWYFDL	1	23	neg	neg	neg	neg
	153-.A04	SF (CD19+ FcRL4-)	1-18	2-15	6	IgG1	7	ARDRHCSGGTCYPYHYGMDV	4	20				
	153-.A06	SF (CD19+ FcRL4-)	4-59	3-10	5	IgG4	5	ARTTTIRGVINWFDP	2	15				
	153-.A07	SF (CD19+ FcRL4-)	1-69	6-19	5	IgM	1	ARDFQRTSTVTRGIAVGSRFDP	4	22				
	153-.A12	SF (CD19+ FcRL4-)	5-51	2-2	3	IgG2	3	ARHLEYPHYVFDF	3	13				
	153-.B07	SF (CD19+ FcRL4-)	4-4	4-17	4	IgM	4	ARGGIWNDYGDFYPYYFDY	1	19				
	153-.C04	SF (CD19+ FcRL4-)	3-23	2-21	6	IgG2	6	AKEDYHFGRVD	3	11				
	153-.C07	SF (CD19+ FcRL4-)	1-18	6-13	5	IgG3	5	ARDGAMGHPDFWQQLVASWFDP	2	22				
	153-.C09	SF (CD19+ FcRL4-)	1-46	5-18	4	IgG1	8	AKSRGYSYGYFDY	2	13				
	153-.C11	SF (CD19+ FcRL4-)	5-51	5-12	6	IgG2	6	ARLPHYDWYYYYAMDV	2	16				
	153-.C12	SF (CD19+ FcRL4-)	4-39	3-16	4	IgG1	12	ARRSVYDANFDF	2	12	neg	neg	neg	neg
	153-.D11	SF (CD19+ FcRL4-)	1-8	3-22	5	IgM	2	ARAPYYYDSSGYYRGWFDP	2	19				
	153-.D12	SF (CD19+ FcRL4-)	3-21	5-24	4	IgA2	8	ARDLVEMATIIGHISY	2	16				
	153-.E12	SF (CD19+ FcRL4-)	4-4	2-15	4	IgG1	10	ARVVSEAAYFDN	1	12	neg	neg	neg	neg
	153-.F02	SF (CD19+ FcRL4-)	3-49	3-10	6	IgG1	12	SRVLRVVWGGRYYCMDV	3	17	neg	neg	neg	neg
	153-.F03	SF (CD19+ FcRL4-)	4-59	3-10	4	IgA1	5	ARVIMFTMVRGVQYYFDY	2	18				
	153-.F05	SF (CD19+ FcRL4-)	4-34	3-10	3	IgA1	5	ARGREVIMVRGVMKGTEAFDI	4	21				
	153-.F06	SF (CD19+ FcRL4-)	4-30	3-16	4	IgA1	8	ARGGREMLTIGGVVLSAFDF	2	20	neg	neg	neg	neg
	153-.F09	SF (CD19+ FcRL4-)	3-9	1-1	4	IgA1	7	VKDITWNRLWVFDS	2	14				
	153-.F10	SF (CD19+ FcRL4-)	4-4	1-14	4	IgG1	7	ARDKGNQPFFDY	2	12	neg	neg	neg	neg
	153-.F11	SF (CD19+ FcRL4-)	1-24	3-22	4	IgA1	14	ATVQNYFDSSGRVTPKSDFDY	2	21				
	153-.F12	SF (CD19+ FcRL4-)	3-48	3-16	4	IgG1	11	AGGRSYDYFDY	1	11	neg	neg	neg	neg
	153-.G02	SF (CD19+ FcRL4-)	3-9	2-15	4	IgM	1	AASYWRGYYFDY	1	12				
	153-.G06	SF (CD19+ FcRL4-)	3-7	3-10	4	IgG1	8	ARGESGGWFGEWVDY	1	15	neg	neg	neg	neg
	153-.G08	SF (CD19+ FcRL4-)	4-61	4-23	5	IgG2	5	ATYAMGYGGKGS	1	12				
	153-.G12	SF (CD19+ FcRL4-)	3-23	5-24	4	IgG1	4	AAPPDGYNSEGYFDY	0	15	neg	neg	neg	neg
	153-.H01	SF (CD19+ FcRL4-)	3-49	3-22	3	IgG2	3	CREEKDYYDRPRDAFDI	4	17				
	153-.H01	SF (CD19+ FcRL4-)	1-2	3-3	5	IgG3	5	ARGIGFNSWSGYPNWFDL	1	18				
	153-.H04	SF (CD19+ FcRL4-)	3-23	3-9	4	IgM	9	ATVSGWGGH	1	9				
	153-.H06	SF (CD19+ FcRL4-)	5-51	3-3	4	IgG1	7	ARHERYYDFWSGYYTEFDY	3	19	neg	neg	neg	neg
	153-.H09	SF (CD19+ FcRL4-)	1-18	3-16	4	IgG1	11	ARDLGFTFGGVMGY	1	14				
	153-.H10	SF (CD19+ FcRL4-)	1-8	3-3	6	IgG4	6	ARGINDFWSDYGMDV	1	15				
	153-.H11	SF (CD19+ FcRL4-)	3-9	6-6	6	IgG2	6	AKDKWKLAGASGGMDV	3	16				
**Patient id**	**Clone**	**cell origin**	**VH**	**DH**	**JH**	**Ig SC**	**VH-mut.**	**VH CDR3 (aa)**	**(+)**	**Length**				
3	423+.A01	SF, CD19+FcRL4+	4-31	4-17	3	IgG1	6	ARGLDTHYGDYELDAFDI	2	18				
	423+.A02	SF, CD19+ FcRL4+	1-69	5-24	6	IgA1	21	TREISAKGANYNYYGMDV	2	18				
	423+.A05	SF, CD19+ FcRL4+	3-49	3-22	3	IgG1	12	ARDRWIVVVPEGGASDI	2	17				
	423+.A06	SF, CD19+ FcRL4+	1-69	5-18	4	IgG2	17	AREEAVDTAMLWYY	1	14				
	423+.A07	SF, CD19+ FcRL4+	1-69	5-24	4	IgG1	18	CAREGLYIATAFFDL	1	15				
	423+.A11	SF, CD19+ FcRL4+	4-30-4	3-22	3	IgA1	27	ATQSLGSSGYYRAFDI	1	16				
	423+.A12	SF, CD19+ FcRL4+	5-10-1	3-22	1	IgA2	22	AKDLLHFPYYYDSSDYYWPAVYFDL	2	25				
	423+.B04	SF, CD19+ FcRL4+	3-30	1-26	4	IgG2	5	ASGPRSGRKDYFDD	3	14				
	423+.B05	SF, CD19+ FcRL4+	1-69	6-19	4	IgA1	13	VRGSSGWNFDH	2	11				
	423+.B06	SF, CD19+ FcRL4+	4-39	3-3	6	IgA1	11	ASSITIFGVVKXXXGMDV	1	18				
	423+.B11	SF, CD19+ FcRL4+	3-49	5-24	4	IgA1	22	AREITSRNGYNHFAY	3	15				
	423+.B12	SF, CD19+ FcRL4+	3-53	5-18	6	IgA1	16	ASGGYSYGLDYYYAMDV	0	17				
	423+.C01	SF, CD19+ FcRL4+	1-46	3-10	2	IgA1	5	ARDQSITMVRGGPPDWNFDL	2	20				
	423+.C05	SF, CD19+ FcRL4+	4-59	1-20	4	IgG2	10	AKSSSPYDWNAPKADY	2	16				
	423+.C07	SF, CD19+ FcRL4+	3-20	6-13	6	IgG1	11	SRDVGSSFPPYYSYAMDV	1	18				
	423+.D05	SF, CD19+ FcRL4+	4-4	3-3	6	IgG3	2	AREGVGSTQGPYYYMDV	1	17				
	423+.D10	SF, CD19+ FcRL4+	4-59	3-16	4	IgG1	6	AAEVMNTDGDVDY	0	13				
	423+.E01	SF, CD19+ FcRL4+	4-39	6-13	4	IgG4	13	ARFPAGYAGSWYVDY	1	15				
	423+.E02	SF, CD19+ FcRL4+	3-21	6-13	4	IgG2	12	ASSPSGPGAAVFDY	0	14				
	423+.E05	SF, CD19+ FcRL4+	1-46	1-1	6	IgG1	4	AKESTATIGTPPEVNYYYGMDV	1	22				
	423+.E08	SF, CD19+ FcRL4+	3-30	3-16	3	IgG1	23	ARETNSYAFDI	1	11				
	423+.F02	SF, CD19+ FcRL4+	4-48	3-10	4	IgG1	14	AGVERDYVSH	2	10				
	423+.F05	SF, CD19+ FcRL4+	4-30-2	3-10	4	IgG1	12	ARVRWGSGSKIDY	3	13				
	423+.F10	SF, CD19+ FcRL4+	1-46	5-18	4	IgA2	17	ARGRGSSYGVTGFDY	2	15				
	423+.G01	SF, CD19+ FcRL4+	1-46	3-10	4	IgG2	17	ARGSGSGSYYNIDY	1	14				
	423+.G03	SF, CD19+ FcRL4+	4-4	3-9	4	IgG1	6	ARDPRRYHILTGHYEGGPSDY	5	21				
	423+.G07	SF, CD19+ FcRL4+	3-30	2-15	6	IgG1	34	AKRTGPVVVSRGGLDV	3	16				
	423+.G10	SF, CD19+ FcRL4+	3-30-3	5-18	5	IgA1	1	AREGGGYSYADNWFDP	1	16				
	423+.G12	SF, CD19+ FcRL4+	3-48	6-19	1	IgG4	6	ARDLPSRGAVAEDFDL	2	16				
	423+.H08	SF, CD19+ FcRL4+	4-59	6-19	3	IgG1	5	AREDPGQTPSGDGPDDAFDI	1	20				
	423+.H09	SF, CD19+ FcRL4+	4-31	6-6	4	IgM	30	AARIASRYFDS	2	11				
	423+.H10	SF, CD19+ FcRL4+	1-69	2-15	3	IgG1	8	ASDVARYCSGGSCYSHAFDI	2	20				
	423+.H11	SF, CD19+ FcRL4+	1-69	5-24	3	IgM	24	ARTGEMATTPNAFDIW	1	16				
	423-.A02	SF (CD19+ FcRL4-)	1-2	1-26	3	IgA1	15	ARGWGAAQVVFDM	1	13				
	423-.A05	SF (CD19+ FcRL4-)	4-34	3-3	4	IgG2	9	ARRTTAYFDFWSDYYFDS	2	18				
	423-.A09	SF (CD19+ FcRL4-)	1-2	1-26	4	IgA1	12	ARGFRSGSYPGY	2	12				
	423-.B01	SF (CD19+ FcRL4-)	3-15	4-23	4	IgG1	18	ATVRRSLSPLKY	3	12				
	423-.B10	SF (CD19+ FcRL4-)	4-31	2-21	6	IgA2	12	ARFRHWYYYIDV	3	12				
	423-.B11	SF (CD19+ FcRL4-)	4-39	3-10	4	IgA1	13	AGLYGDLFPGVMRYFDP	1	17				
	423-.C02	SF (CD19+ FcRL4-)	3-23	3-10	4	IgA1	19	ANAGTGYLPFDY	0	12				
	423-.C03	SF (CD19+ FcRL4-)	3-33	3-10	6	IgM	4	ASRGGVGGYYVKDYGMDV	2	18				
	423-.C04	SF (CD19+ FcRL4-)	5-51	3-22	6	IgG1	2	ARLRYYYDSSGYYYMNNYYYYYMDV	2	25				
	423-.C05	SF (CD19+ FcRL4-)	3-23	5-18	4	IgG2	27	AKDVVDSVMGLPFDY	1	15				
	423-.C09	SF (CD19+ FcRL4-)	3-33	3-16	5	IgA1	3	AREGDLIPDRFDP	2	13				
	423-.C12	SF (CD19+ FcRL4-)	1-18	2-2	6	IgM	5	SRVGRVPDAVRYYFYGDV	3	18				
	423-.D01	SF (CD19+ FcRL4-)	1-69	5-12	5	IgA1	29	ARDRRGGNRRRENWFDP	6	17				
	423-.D02	SF (CD19+ FcRL4-)	3-43	6-13	4	IgG2	9	AAAPGRRFDY	2	10				
	423-.D03	SF (CD19+ FcRL4-)	4-31	2-21	4	IgG1	16	ARGSGSGYDLAYCGGDCYFLLDK	2	23				
	423-.D06	SF (CD19+ FcRL4-)	4-30-4	3-22	4	IgG1	3	AAYPGSYYDNSGRHLISPPFDN	2	22				
	423-.E02	SF (CD19+ FcRL4-)	3-48	5-12	3	IgG2	20	ARGGYSGYLLTHDAFDI	2	17				
	423-.E07	SF (CD19+ FcRL4-)	3-30	3-3	4	IgG1	12	ANEVDFWSGYYDY	0	13				
	423-.E08	SF (CD19+ FcRL4-)	3-49	3-10	6	IgG1	18	SRVDRVVRGGRYYYYCMDV	4	19				
	423-.E12	SF (CD19+ FcRL4-)	4-61	6-19	4	IgM	6	ARVPRGWYYIDY	2	12				
	423-.F03	SF (CD19+ FcRL4-)	3-23	2-8	4	IgG1	10	AKPLVYARLYFDY	2	13				
	423-.F04	SF (CD19+ FcRL4-)	3-33	6-13	5	IgG1	15	ARDPPTSQYSSTWWTDRGFDH	3	21				
	423-.F06	SF (CD19+ FcRL4-)	3-11	2-21	4	IgG1	22	AREGPIVVVPVV	1	12				
	423-.F07	SF (CD19+ FcRL4-)	1-2	3-10	4	IgG3	14	ASGVNADGEGGPPTVGY	0	17				
	423-.F10	SF (CD19+ FcRL4-)	5-10-1	2-15	3	IgG1	10	AKAASRFDTFDI	2	12				
	423-.G03	SF (CD19+ FcRL4-)	3-33	6-19	4	IgG1	32	ARDRQWLLDY	2	10				
	423-.G05	SF (CD19+ FcRL4-)	3-9	3-16	5	IgM	1	AKGGWITLGSWFDP	1	14				
	423-.H03	SF (CD19+ FcRL4-)	1-46	6-19	4	IgG1	18	ARVVTDTAGWYHFDY	2	15				
	423-.H04	SF (CD19+ FcRL4-)	1-46	2-2	4	IgG3	12	ARGRLPAAIRIDFDY	3	15				
	423-.H06	SF (CD19+ FcRL4-)	3-30-3	6-19	3	IgG1	8	AKDGKAVDGFSGVLEM	2	16				
	423-.H07	SF (CD19+ FcRL4-)	4-31	3-10	4	IgG1	29	AGVERDYVSH	2	10				
	423-.H11	SF (CD19+ FcRL4-)	4-30-4	5-18	6	IgG1	19	ARERSYGRQYHYGMDV	4	16				
**Patient id**	**Clone**	**cell origin**	**VH**	**DH**	**JH**	**Ig SC**	**VH-mut.**	**VH CDR3 (aa)**	**(+)**	**Length**				
2	03+.A02	ST, CD19+ FcRL4+	4-59	6-13	1	IgA1	1	ARYFFGGMSAAGSYFQH	2	17				
	03+.A03	ST, CD19+ FcRL4+	5-51	3-22	5	IgG2	26	ARLSGYYDSSGYYYPYNWFDS	1	21				
	03+.A09	ST, CD19+ FcRL4+	5-51	3-10	3	IgG1	19	VRHILWFGESDSFDI	2	15				
	03+.B01	ST, CD19+ FcRL4+	1-3	2-21	4	IgG3	25	ARSHQPYILLAGRPGV	3	16				
	03+.B02	ST, CD19+ FcRL4+	3-33	4-17	6	IgA1	25	CAGDYITRPNFSYYYYGMDV	1	20				
	03+.B03	ST, CD19+ FcRL4+	3-30-3	5-12	4	IgG1	1	AKYHVDIVATSLEYFDY	2	17				
	03+.B04	ST, CD19+ FcRL4+	3-21	3-3	5	IgA1	4	ARVWEDWFDP	1	10				
	03+.B05	ST, CD19+ FcRL4+	3-48	5-24	6	IgG1	13	AKDQPHGHIYYGLDV	3	15				
	03+.B06	ST, CD19+ FcRL4+	4-34	3-22	4	IgM	14	ARDREYYDSRGYYSFDY	3	17				
	03+.B09	ST, CD19+ FcRL4+	1-18	3-22	4	IgG1	13	AREFPYDSSGYFPGGGDY	1	18				
	03+.B09	ST, CD19+ FcRL4+	4-34	6-13	4	IgG1	9	ARGPPRAVPGTARRRYFDS	5	19				
	03+.C02	ST, CD19+ FcRL4+	3-33	2-15	5	IgG1	28	ARHGRGPSSWYDF	3	13				
	03+.C03	ST, CD19+ FcRL4+	1-69	2-15	6	IgG1	4	ARGYCSGGSCFDHYYYYGMDV	2	21				
	03+.C10	ST, CD19+ FcRL4+	1-69	1-26	4	IgG1	16	ARGFQVGTITGFDY	1	14				
	03+.C11	ST, CD19+ FcRL4+	3-20	2-2	4	IgA1	11	AKSLIGVESSFDS	1	13				
	03+.C12	ST, CD19+ FcRL4+	3-30-3	3-22	4	IgG1	10	ARGKDYYDSTGYYWGILDD	2	19				
	03+.D02	ST, CD19+ FcRL4+	3-21	1-26	4	IgG1	2	ARDRRVPYIVGATDFDY	3	17				
	03+.D04	ST, CD19+ FcRL4+	3-33	3-10	6	IgA2	31	ARGPSGMFGDLSPYFHYGVDV	2	21				
	03+.D06	ST, CD19+ FcRL4+	3-33	2-15	6	IgA1	24	ARDREAATPKYGMDV	3	15				
	03+.D07	ST, CD19+ FcRL4+	3-74	6-19	2	IgG1	6	AREVEQWLEHGVLWYFDL	2	18				
	03+.D08	ST, CD19+ FcRL4+	1-3	4-17	6	IgG3	13	AGDYITRPNFSYYYYGVDV	1	19				
	03+.F01	ST, CD19+ FcRL4+	4-39	5-24	3	IgG1	10	ARDREMGHQGIFDI	3	14				
	03+.F03	ST, CD19+ FcRL4+	1-3	2-2	6	IgG4	9	SRDRSISWDGDGMDVW	2	16				
	03+.F04	ST, CD19+ FcRL4+	4-39	2-8	4	IgG1	13	ARDKGNQPFFDY	2	12				
	03+.F05	ST, CD19+ FcRL4+	3-33	3-10	6	IgA1	29	ASRGGVGGYYVKDYGVDV	2	18				
	03+.F07	ST, CD19+ FcRL4+	4-4	3-3	6	IgG1	6	ARVSSAKTTFGVTTTWGGMDV	2	21				
	03+.F08	ST, CD19+ FcRL4+	3-20	4-17	4	IgA1	4	ARGGGPGDKVRGDY	3	14				
	03+.G03	ST, CD19+ FcRL4+	3-66	2-2	6	IgA1	1	ARGGTISRYYYFGMDV	2	16				
	03+.G07	ST, CD19+ FcRL4+	1-69	6-19	6	IgA1	19	ARGAVAGRHYYFGLDV	3	16				
	03+.G08	ST, CD19+ FcRL4+	1-69	6-13	5	IgG2	4	TREAAAAGRNNWFDP	2	15				
	03+.G12	ST, CD19+ FcRL4+	1-3	2-8	4	IgG2	13	ARSHQPYILLAGTPGD	2	16				
	03+.H01	ST, CD19+ FcRL4+	4-4	3-22	4	IgG4	18	CACRYLGLDY	1	10				
	03+.H04	ST, CD19+ FcRL4+	4-34	4-23	6	IgG1	9	CARVPEVVTPRYYYYFGLDV	2	20				
	03-.A01	ST (CD19+ FcRL4-)	3-9	2-15	4	IgG3	33	VKDGGAGGANTFDH	2	14				
	03-.A02	ST (CD19+ FcRL4-)	1-69	2-21	6	IgG1	29	ASKGGDSVGYHYYMDV	2	16				
	03-.A03	ST (CD19+ FcRL4-)	3-23	6-13	4	IgA1	7	AKGPYSSSWYGAPFDY	1	16				
	03-.A04	ST (CD19+ FcRL4-)	1-69	5-24	3	IgG1	22	ARDREMGHQGIFDI	3	14				
	03-.A06	ST (CD19+ FcRL4-)	1-69	6-13	4	IgG1	4	ARARIAAAGNPGSFDY	2	16				
	03-.A08	ST (CD19+ FcRL4-)	4-59	3-10	4	IgG1	4	ARDSSYEDSVYFDY	1	14				
	03-.A12	ST (CD19+ FcRL4-)	1-18	2-2	4	IgG4	4	ARDEFQLPDY	1	10				
	03-.B06	ST (CD19+ FcRL4-)	3-23	1-26	6	IgG1	28	AKSWAILQFEPLYGMDV	1	17				
	03-.B11	ST (CD19+ FcRL4-)	3-53	3-16	6	IgG1	15	TRERPHEYVWGSFRRHYGMDV	6	21				
	03-.C01	ST (CD19+ FcRL4-)	3-49	4-17	5	IgA1	29	TIVFPELPRVPLP	1	13				
	03-.C03	ST (CD19+ FcRL4-)	3-23	3-22	1	IgG2	14	AKDRAGNNSGYYYVGEYFQH	3	20				
	03-.C05	ST (CD19+ FcRL4-)	4-34	2-8	4	IgG1	4	ARGRETYCAAGVCSSKGKRPDYFDY	5	25				
	03-.C08	ST (CD19+ FcRL4-)	3-30-3	3-3	4	IgA2	31	AREFEHFGSGYFPVDY	2	16				
	03-.C09	ST (CD19+ FcRL4-)	3-21	3-22	3	IgG2	30	ARDRFPSDDYDGPEGFDL	2	18				
	03-.D04	ST (CD19+ FcRL4-)	4-30-4	3-3	4	IgG1	29	ARASTLEWSYGSFDY	1	15				
	03-.D05	ST (CD19+ FcRL4-)	3-23	3-22	4	IgG1	19	AKADYSDSSGYKDY	2	14				
	03-.D07	ST (CD19+ FcRL4-)	3-74	3-3	5	IgG1	13	ASSYRIWSFDP	1	11				
	03-.D08	ST (CD19+ FcRL4-)	3-23	2-8	4	IgA1	1	AKPMVYARLYFDY	2	13				
	03-.D11	ST (CD19+ FcRL4-)	3-23	3-9	4	IgG1	19	AKDFTRYFDWLLRDLFDY	3	18				
	03-.E07	ST (CD19+ FcRL4-)	3-23	5-24	4	IgG1	15	ARDRMACDY	2	9				
	03-.E08	ST (CD19+ FcRL4-)	3-11	3-22	4	IgG2	18	ARDLTGMNSDSSGYYSDY	1	18				
	03-.E11	ST (CD19+ FcRL4-)	3-23	3-10	6	IgG1	22	AKAGQSPDMVRGVIRWGPKPEPKNSYYGMDV	5	31				
	03-.F08	ST (CD19+ FcRL4-)	3-30	1-26	4	IgA1	23	AKGVGALQAGLGSGVYYFNY	1	20				
	03-.F10	ST (CD19+ FcRL4-)	4-39	5-24	5	IgM	25	ARRGHYVWFDP	3	11				
	03-.G07	ST (CD19+ FcRL4-)	3-20	3-22	4	IgM	8	ARGPPYYISSGYYFSFDS	1	18				
	03-.G11	ST (CD19+ FcRL4-)	3-7	3-9	5	IgA1	6	ARTPGTFHTHNWFDP	3	15				
	03-.H04	ST (CD19+ FcRL4-)	4-31	3-10	5	IgG1	25	ARVVPAHHFPSGGHSSSAFNWFDP	4	24				
	03-.H10	ST (CD19+ FcRL4-)	3-33	3-3	4	IgA1	15	TRSLGYCTRSTCYSHEHYDH	5	20				
**Patient id**	**Clone**	**cell origin**	**VH**	**DH**	**JH**	**Ig SC**	**VH-mut.**	**VH CDR3 (aa)**	**(+)**	**Length**				
6	508+.A05	SF (CD19+ FcRL4-)	1-8	2-15	5		18	ARGHGHCSDSGCFNNWFDP	3	19				
	508+.A07	SF (CD19+ FcRL4-)	3-66	3-9	4		15	ARSPTGYDILTGPFDY	1	16				
	508+.A10	SF (CD19+ FcRL4-)	3-7	3-10	6		32	ARDNARAWFSHYYYGMDV	3	18				
	508+.B03	SF (CD19+ FcRL4-)	3-11	3-10	6		0	ARDLRYYGSGMYYTYYYYGMDV	2	22				
	508+.D05	SF, CD19+ FcRL4+	3-33	3-10	6		28	ASRGGVGGYYVKDYGMDV	2	18				
	508+.D11	SF (CD19+ FcRL4+)	3-11	3-10	6		33	ARMWFGDDHYYYGLDI	2	16				
	508+.E03	SF (CD19+ FcRL4+)	4-31	3-3	4		18	ARGQRGAILVHGYIPFFDF	3	19				
	508+.E06	SF, CD19+ FcRL4+	3-30	6-6	4		20	AKDPHSSSLISPPLFGY	2	17				
	508+.E09	SF (CD19+ FcRL4+)	3-74	3-3	1		19	ARVFKGWSSWYQGSPSEYFQH	3	21				
	508+.E10	SF (CD19+ FcRL4+)	1-69	5-18	5		21	ARGRTYTYGPMRWFDP	3	16				
	508+.E11	SF (CD19+ FcRL4+)	3-48	6-13	5		28	ARGQGRIEYNWFDL	2	14				
	508+.F02	SF, CD19+ FcRL4+	4-34	4-11	6		12	ARPTHSTVTMWYFGMDV	2	17				
	508+.F04	SF, CD19+ FcRL4+	3-30	3-10	5		19	ARDPINYYGSGSYSWNWIDP	1	20				
	508+.F09	SF, CD19+ FcRL4+	3-11	3-9	6		3	ASPSGNPNPFTMDV	0	14				
	508+.F11	SF, CD19+ FcRL4+	3-15	3-16	2		10	AREVTPHWYFDL	2	12				
	508+.F12	SF, CD19+ FcRL4+	1-46	2-15	4		12	ARGGPFTNPLCSASTCYYFDS	1	21				
	508-.B04	SF (CD19+ FcRL4-)	3-7	2-21	4		17	ARAADYGPVAGLFEY	1	15				
	508-.B09	SF (CD19+ FcRL4-)	1-8	3-10	4		17	AFHLGEYGSGSYYDLDY	1	17				
	508-.B10	SF (CD19+ FcRL4-)	3-21	5-12	5		28	ARVWVTGAAIFGDNWFDP	1	18				
	508-.C04	SF (CD19+ FcRL4-)	1-8	3-3	4		29	ARAAVDFWSGYHLEY	2	15				
	508-.C07	SF (CD19+ FcRL4-)	1-24	2-15	4		9	AIMGALYCSGGDCYLRGAGEFDY	1	23				
	508-.D06	SF (CD19+ FcRL4-)	3-48	2-8	5		14	ARGLGRLCGADNCYNWFDP	2	19				
	508-.D08	SF (CD19+ FcRL4-)	3-33	2-2	6		13	ARARYSSSSYGMDV	2	14				
	508-.D10	SF (CD19+ FcRL4-)	3-23	6-19	4		7	AREGIPVAGTDY	1	12				
	508-.E12	SF (CD19+ FcRL4-)	3-15	3-3	4		13	TAYRITPFGVLTGFGERPVDY	2	21				
	508-.F03	SF (CD19+ FcRL4-)	4-61	2-2	4		17	ARIKGGYCSYTNCKRPVPFDY	4	21				
	508-.F08	SF (CD19+ FcRL4-)	4-30-4	5-12	4		4	ATAPRSPTGYDSFYLDS	1	17				
	508-.G05	SF (CD19+ FcRL4-)	3-48	5-18	3		17	ARGRKGYSYDAFDI	3	14				
	508-.G08	SF (CD19+ FcRL4-)	3-30	4-17	5		5	AQDRVAALTRGGLGWFDP	2	18				
	508-.G09	SF (CD19+ FcRL4-)	1-69	4-11	4		4	ARELYSNYFF	1	10				
	508-.H07	SF (CD19+ FcRL4-)	4-34	7-27	4		4	ARLRPRLRGDLDS	4	13				
	508-.H08	SF (CD19+ FcRL4-)	1-18	6-19	4		4	ARTTGGDSGWFDHMDF	2	16				
	508-.H11	SF (CD19+ FcRL4-)	3-43	3-22	6		36	AKGLRKTDVYYDSSGFGYYYGMDV	3	24				
	508-.H12	SF (CD19+ FcRL4-)	1-24	3-9	4		4	ATENRFRHFWYGFDF	3	15				
**Patient id**	**Clone**	**cell origin**	**VH**	**DH**	**JH**	**Ig SC**	**VH-mut.**	**VH CDR3 (aa)**	**(+)**	**Length**				
4	531+.A01	SF (CD19+ FcRL4-)	3-21	1-1	4		15	ARCRPGSTSPEF	2	12				
	531+.B07	SF (CD19+ FcRL4+)	3-74	3-10	4		28	ARERSRIIDY	3	10				
	531+.B08	SF (CD19+ FcRL4+)	4-39	4-17	2		6	TRQWGSDYGDYWYFDL	1	16				
	531+.B09	SF (CD19+ FcRL4+)	4-39	3-3	6		11	SRDQRITILGVVSVWFGMDV	2	20				
	531+.C02	SF (CD19+ FcRL4+)	1-18	2-2	4		15	ARALLDGYCTGSSCAVGSMDY	1	21				
	531+.C07	SF (CD19+ FcRL4+)	3-33	6-19	2		19	AGSLSSGWHGNRYFDL	2	16				
	531+.C08	SF, CD19+ FcRL4+	4-34	6-6	4		17	AKGSTSSLYRHTMPYQY	3	17				
	531+.C12	SF (CD19+ FcRL4+)	3-9	1-1	4		22	ARDAKYYFDY	2	10				
	531+.D03	SF (CD19+ FcRL4-)	4-34	1-26	3		5	ARSWELLLGAFDI	1	13				
	531+.G01	SF, CD19+ FcRL4+	1-69	6-13	6		28	ATAGYTSRWNPSFYHGLDV	2	19				
	531+.G02	SF, CD19+ FcRL4+	1-3	3-10	4		23	ARDYGSGNSGYFDY	1	14				
	531+.G03	SF, CD19+ FcRL4+	3-33	4-17	4		3	VTDYGDYVELGY	0	12				
	531+.G04	SF, CD19+ FcRL4+	3-15	6-19	6		15	ARGWLEPFYYYGVDV	1	15				
	531+.G11	SF, CD19+ FcRL4+	3-11	3-10	5		15	ARDLLVHGVAISNWFDP	2	17				
	531+.H07	SF, CD19+ FcRL4+	4-4	5-12	6		19	ARYSGFYYHYGMDV	2	14				
	531-.D10	SF (CD19+ FcRL4-)	3-23	3-3	6		25	AKGGSGAFWSGYYKNYYYYYMDV	2	23				
	531-.D11	SF (CD19+ FcRL4-)	1-46	3-3	6		14	ARVTFESNDFGPDHFYYVLDV	2	21				
	531-.D12	SF (CD19+ FcRL4-)	3-69	1-26	4		3	ARHNGSYKKGYYFDY	4	15				
	531-.E01	SF (CD19+ FcRL4-)	3-48	3-16	4		22	AGGRSYDYFDY	1	11				
	531-.E02	SF (CD19+ FcRL4-)	3-23	4-23	4		2	AKEVQTEGGFDY	1	12				
	531-.E03	SF (CD19+ FcRL4-)	4-4	2-15	4		23	ARVVSEAAYFDN	1	12				
	531-.E09	SF (CD19+ FcRL4-)	3-11	5-18	4		15	ARRGGYSYRKDYFDS	4	15				
	531-.E12	SF (CD19+ FcRL4-)	3-23	5-18	4		4	AKDKWEGAMNPHYFDF	3	16				
	531-.F02	SF (CD19+ FcRL4-)	3-33	3-16	5		1	ARETFERIRLGEPNWFDP	3	18				
	531-.F03	SF (CD19+ FcRL4-)	4-34	3-3	4		25	TRDLSRKIFGVVKPAFYFDY	4	20				
	531-.F05	SF (CD19+ FcRL4-)	3-48	3-22	4		5	AREGEGDLYYYDSSGYYYL	1	19				
	531-.F07	SF (CD19+ FcRL4-)	1-2	3-22	3		3	ASKKEGVLPLDPFDI	2	15				
	531-.G03	SF (CD19+ FcRL4-)	4-31	4-11	6		2	GRTLATVPMDV	1	11				
	531-.G11	SF (CD19+ FcRL4-)	3-23	6-19	4		9	AKGSVAGPFDY	1	11				
	531-.H07	SF (CD19+ FcRL4-)	1-18	3-3	3		9	ARAEGAVTIDDAFDI	1	15				
	531-.H08	SF (CD19+ FcRL4-)	4-31	3-10	3		12	ARDGAGRDAFDM	2	12

**Table 3:** Number of sequences and recombinant monoclonal antibodies generated from FcRL4+ and FcRL4- B cells respectively.

**Patient ID**	**Sample**	**Diagnosis**	CCP2	RF	**FcRL4**	**Number of sorted 96-well plates**	**Number of retrieved sequences**	**Number of ab cloned**	**Number of ab. expressed**
1	ST	RA	pos	pos	positive	1	59	34	26
					negative	1	33	23	17
2	ST	RA	pos	pos	positive	1	33	ND	ND
					negative	1	28	ND	ND
3	SF	RA	neg	neg	positive	1	33	ND	ND
					negative	1	32	ND	ND
4	SF	RA	neg	neg	positive	1	15	ND	ND
					negative	1	16	ND	ND
5	SF	RA	pos	pos	positive	1	30	22	10
					negative	1	33	25	10
6	SF	RA	na	pos	positive	1	16	ND	ND
					negative	1	18	ND	ND

## 2 Experimental design, materials and methods

More detailed information can be found in Ref. [1].

### 2.1 Experimental design

FcRL4+ and FcRL4- B cells were sorted from synovial fluid and tissue from RA patients. Synovial fluid derived B cells were analysed for their gene expression profile by RNAseq. Immunoglobulin variable region genes from single sorted B cells were sequenced and expressed as components of recombinant monoclonal antibodies. These were investigated for their reactivity with autoantigens. Amplification of sections of Ig constant regions was used to identify Ig isotype usage.

### 2.2 Materials and methods

Cells from 4 SF and 2 ST samples were stained for CD19 and FcRL4 and sorted either as single cells or as cell populations. Individual IgH and IgL chain gene rearrangements were PCR-amplified independently. For identification of Ig isotypes, amplification of IgH chains with reverse primers specific for the constant regions of all human Ig classes and sequencing was used. Cloning of the Ig genes into expression vectors and antibody production and purification were performed. Antibody reactivity against citrullinated peptides was determined by ELISA. For gene expression analysis RNA sequencing was carried out on sorted FcRL4+ and FcRL4- B cells from 4 SF samples. Preamplification prior to Illumina Truseq library preparation was performed using the SMARTer amplification using olig(dT) primed reactions. Library prep was carried out using the Illumina TruSeq Stranded prep kit and sequenced on the Illumina HiSeq. 2000/2500 platform. Data were analysed as detailed in [1].
