# Supplementary material for: Immunoglobulin characteristics and RNAseq data of FcRL4+ B cells sorted from synovial fluid and tissue of patients with rheumatoid arthritis
Source: Data Brief. 2017 Jun 7;13:356–70. doi: 10.1016/j.dib.2017.06.009 (PMC5480816; doi:10.1016/j.dib.2017.06.009)
Supplement: Supplementary file 1 — Supplementary material [file mmc1.docx]

The authors have reported in the primary article that there is no conflict of interest:

Amara K, Clay E, Yeo L, Ramsköld D, Spengler J, Sippl N, Cameron JA, Israelsson L,Titcombe PJ, Grönwall C, Sahbudin I, Filer A,Raza K, Malmström V, Scheel-Toellner D.

B cells expressing the IgA receptor FcRL4 participate in the autoimmune response in patients with rheumatoid arthritis. J Autoimmun. 2017. pii: S0896-8411(16)30396-1. doi: 10.1016/j.jaut.2017.03.004 [Epub ahead of print]
